# Participants' perspective on maintaining behaviour change: a qualitative study within the European Diabetes Prevention Study

**DOI:** 10.1186/1471-2458-8-235

**Published:** 2008-07-10

**Authors:** Linda Penn, Suzanne M Moffatt, Martin White

**Affiliations:** 1 Public Health Research Programme, Institute of Health and Society, Newcastle University, Newcastle upon Tyne, UK

## Abstract

**Background:**

The European Diabetes Prevention Study (EDIPS) is an RCT of diet and exercise interventions in people with impaired glucose tolerance. We undertook a qualitative study, nested within the EDIPS in Newcastle-upon-Tyne, UK, aiming to understand the experience of participants who maintained behaviour change, in order to inform future interventions.

**Methods:**

Participants were purposively sampled, according to success criteria for diet and physical activity change maintenance, and invited to attend individual semi-structured interviews. Fifteen participants completed an interview and reflected on their experience over three to five years. We used the Framework method to analyse the transcribed data.

**Results:**

Main themes were identified as factors that help (props) and those that hinder (burdens) behaviour change maintenance at different organisational levels: individual (both physical and psychological), social and environmental. Pre-existing physical conditions (such as arthritis) and social demands (such as caring for an ageing relative) hindered, whereas the benefits of becoming fitter and of having social and professional support helped, participants in maintaining behaviour change. Participants' long term experiences highlighted the salience of the continuous change in their physical, social and environmental conditions over time.

**Conclusion:**

The construct of props and burdens facilitates a holistic view of participants' behaviour. Efforts to encourage behaviour change maintenance should take account of context and the way this changes over time, and should include strategies to address these issues. The experience of participants who maintain behaviour change highlights the challenges for the wider implementation of diabetes prevention strategies.

**Trial Registration:**

(ISRCTN 15670600)

## Background

Maintaining behaviour change is important for the effectiveness of lifestyle interventions to prevent type 2 diabetes, but it requires continual effort and the ability to adapt to changing circumstances. Successful intervention development requires evidence from long-term studies to investigate change maintenance. A staged approach to the development and evaluation of complex interventions has been promoted [1-3]. This approach advocates combining quantitative and qualitative research methods to help us to understand and refine interventions so that wider implementation can be both effective and efficient [4].

The prevalence of type 2 diabetes is increasing and there are causal links with obesity,[5] diet[6] and lack of exercise [7]. The UK National Service Framework for diabetes has identified the prevention of type 2 diabetes as a priority [8]. Impaired glucose tolerance (IGT) is considered to be a stage in the development of type 2 diabetes and studies have shown that progression to diabetes can be prevented or delayed by changes in the lifestyles of those at risk [9-12]. The Finnish Diabetes Prevention Study (DPS) showed a 58% reduction in diabetes incidence following lifestyle intervention. The European Diabetes Prevention Study (EDIPS) extends the DPS to different European populations and uses the same study design which has been described in detail [13]. One hundred and two participants with IGT were recruited to the EDIPS in Newcastle-upon-Tyne UK and randomised to a lifestyle intervention group or usual care control group. The diagnosis of IGT was on the basis of two oral glucose tolerance tests and this was an EDIPS inclusion criterion. The intervention included individual motivational interviewing, delivered by a physiotherapist and a dietician at three month intervals, aimed at reducing total food energy and fat intake, and at increasing activity [14]. The control group received general advice at the start of the trial. Both groups attended an annual clinical review, which included an oral glucose tolerance test and measurement of height and weight. They also completed a three day food and a three day activity diary each year throughout the five year study period.

The dimensions of achieving and maintaining lifestyle change are complex, including social, psychological [15] and educational [16] aspects. The EDIPS intervention was based on the trans-theoretical model of health behaviour change (TTM)[17].

Many studies have reported failure to achieve intended behaviour change goals [18].

Earlier qualitative studies relating to the management of diet and exercise in people with diabetes have used a realist approach where the interview data is used to access people's beliefs[19]. In some studies researchers have sought an explanation for compliance or non compliance with the advised regime in order to identify modifiable factors affecting these outcomes [20]. Criteria to define compliance can be arbitrary and are usually general rather than individual.

More recently, in qualitative studies of health issues, there has been much interest in considering the dialogue within interviews from a discursive psychological perspective [21]. The focus in this approach is on the interview discourse itself and the 'action orientation' of the participants. How people choose to present themselves in dialogue is an extra layer in understanding how people attempt behaviour change. Discursive psychology can provide insight into the meaning structure of dialogue.

We undertook a qualitative study embedded within the EDIPS RCT in Newcastle upon Tyne to explore the maintenance of behaviour change with a view to informing and improving intervention design. We had access to the individual process data from the trial annual food and activity diaries over three to five years and therefore had the opportunity to define success, for our qualitative study, in terms of change achieved in process outcomes rather than compliance with an externally applied target criterion.

## Methods

### Design and participants

Ethical approval was given by the Newcastle-upon-Tyne NHS Local Research Ethics Committee and research governance approval was received from Newcastle PCT. All participants gave written informed consent. We used individual data from the EDIPS in Newcastle (ISRCTN 15670600) to sample purposively, according to three success criteria in behavioural process outcomes: increased activity, calorie reduction and fat reduction. This data was obtained from the individual activity and food diaries that the participants completed annually. The success criteria in each category were norm referenced, based on individual percentage beneficial change compared with baseline. (For example reduction of fat consumed, calorie equivalent, from 40% to 30% is a 25% beneficial change). Participants who maintained change in one or more categories for at least two years were selected. Sampling details are given in Table 1. The sample included both intervention and control group participants. The analysis of control group success was important for intervention development. To provide a representative profile we considered gender, EDIPS group and length of participation in the EDIPS (3, 4 or 5 years). We invited 25 people who fulfilled the success criteria in one or more categories and, to cover a range of experience, a further three randomly selected participants who had not achieved success. Fifteen people accepted and completed interviews lasting 45 to 60 minutes. The sample size and decision to focus primarily on success were resource driven and objective criteria for sample selection were important. LP was the EDIPS trial researcher at the time of this qualitative study and had some knowledge of the participants, although she was not involved in delivering the intervention. Participant characteristics are shown in Table 2.

**Table 1:** Data collection: sampling frame, success criteria and invitation strategy for sampling.

**Sampling frame**: The qualitative study was nested in the EDIPS Newcastle trial. The first recruit to the RCT was in Sept 2000 and the last recruit was in Sept 2002. Participants were all aged over 40 years and Caucasian with BMI > 25 and with IGT diagnosed on the basis of 2 OGTTs	
**Data collection**. For each measurement criterion we compared the individual baseline scores with subsequent years.	**Success criteria**. Criteria measured: *'relative to the individual baseline score, maintained for two or more years.'*
The activity diaries covered 24 hours for three days and activity was scored in half hour divisions on a MET score scale.	Increased activity: (n = 20, Control = 7, Intervention = 13) *'More than 10% increase in activity.'*
The food diaries covered three days and were analysed using Microdiet software to determine total calories consumed.	Calorie reduction: (n = 13, Control = 5, Intervention = 8) *'More than 20% calorie reduction.'*
Calorie intake attributable to fat consumption was calculated from the food diary analysis.	Fat reduction: (n = 18, Control = 9, Intervention = 9) *'More than 5% fat reduction (calorie equivalent).'*

**Invitation strategy** A 'pool' of potential participants was selected on the basis of the diary data success criteria (n = 38). We excluded seven for specific reasons (e.g. 1. her husband had just died, 2. she had just had extensive surgery, 3. he had already completed five years in the trial and had requested no further study invitations) and we added three, randomly selected from the trial participants who had not achieved success as defined to broaden the range of experience considered. We then invited participants in groups of five. After each group acceptance we looked at the demographic data to maintain an overall sample balance. For example: if 3 females accepted in the first group and 2 males declined, we would invite proportionately more males for the next group so that the demographics (age, gender, trial group, success measure and trial years completed) of our interviewed sample reflected the demographics of the sample pool.	

**Table 2:** Participant characteristics

**Participant Number**	**Gender**	**Age** Mean = 64	**Group** I: Intervention C: Control	**Year of follow up**	**Success** f. fat reduction c. calorie reduction a. activity increase n. no success	**Employment status** 1. retired 2. working 3. unable to work	**Marital status** 1. married or co-habiting 2. single 3. separated or divorced 4. widowed	**Standard occupational classification **(previous if not now working) 2. professional 3. associate professional 4. administrative 5. skilled trade 8. process operatives 9. elementary occupations
1	M	67	I	5	f	1	1	5
2	F	63	I	5	a	1	4	9
3	M	66	C	3	a	1	4	9
4	M	51	I	4	acf	2	2	9
5	M	72	I	4	cf	1	1	8
6	M	74	C	3	cf	1	1	9
7	M	67	I	3	acf	1	1	2
8	M	72	I	3	a	1	1	2
9	F	57	C	4	f	3	1	2
10	F	60	I	3	af	1	1	2
11	F	47	C	4	af	2	1	3
12	F	72	C	5	f	1	3	5
13	F	68	I	5	c	1	3	4
14	M	70	C	4	c	1	1	
15	F	56	I	3	n	3	1	9

### Data collection

Semi-structured interviews were undertaken by LP in a private, quiet room in Newcastle University. The initial topic guide was prepared from the literature and the experience of the trial professionals. Flexibility in the interview structure allowed participants to discuss issues of interest to them. The topic guide was phrased more as 'areas to discuss' rather than direct questions. This was to allow people to follow there own trains of thought. For example we might ask about eating out. If they replied that they didn't often eat out we would follow this up with questions about entertaining and visiting friends. This would expand the definition of eating out and also provide the opportunity to revisit the topic area.

The topic guide was refined and expanded as the interviews progressed. The interviews were recorded, transcribed and imported into NVivo data analysis software. After the early interviews the co-authors discussed and agreed emerging themes to develop in subsequent interviews.

### Analysis

The Framework approach to data analysis was used, a content analysis technique widely used in qualitative research [22]. The research aim was to develop an understanding of behaviour change maintenance with a view to improving intervention design. In the analysis we sought to identify themes to highlight modifiable influences on behaviour. Transcripts were read, checked and imported into the NVivo computer program,[23] codes, themes and conceptual constructs were developed and thematic charts constructed by LP [24]. The co-authors met regularly to discuss the coding concepts and the development of the themes with reference to the transcript data. The greater familiarisation with the data, which this process required, led to the development of further categories to better represent the meaning inherent in the transcripts. The behaviour change literature was revisited to triangulate and check for sensitivity and completeness in identifying themes, and consideration was given to presenting the data to adequately convey the results of the analysis in an interesting and meaningful way that is easily accessible [24].

From the identified themes (first order constructs) the data were further analysed using an empirical phenomenology approach as described by Aspers [25], which is about studying consciousness as it is experienced and trying to understand another person's subjective world as it is for that person. This further analysis, with reference to commonly used health behaviour theories and with some appreciation of the participants' meaning structure from the EDIPS trial experience, led to the development of second order constructs [17]. The process by which behaviour change theory, the empirical data with first order constructs and the participants' province of meaning were considered with reflection to elucidate three, more abstract, second order constructs is elaborated in the discussion section.

## Results

**Figure 1:**
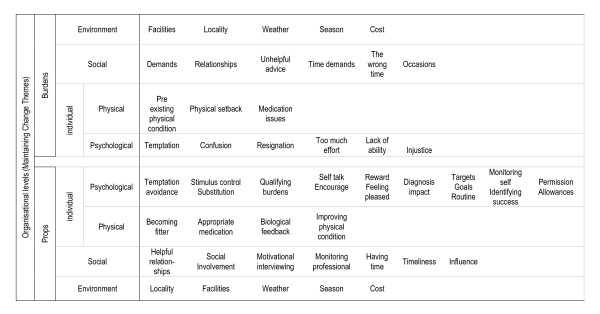
Themes for change maintenance in organisational levels (props and burdens).

We identified categories for individual (physical and psychological), social and environmental themes and we identified contrasting facets within these. For example, there was temptation, "I like chocolate," and temptation avoidance strategies, "I don't have chocolate in the house." We conceptualised this contrast as, 'props and burdens'. We used props to categorise anything that helped, and burdens to categorise anything that hindered, in maintaining a healthy lifestyle. The word burden was used specifically to emphasise the ongoing nature of the problems that people had. The long follow up period in the EDIPS trial, with participants able to reflect on their experience over three to five years, allowed the consideration of change maintenance over time. Figure 1 summarises the props and burdens we identified and shows these in organisational levels in line with health promotion theory [26]. The key themes are described and discussed in detail below.

From further analysis of these first order constructs (props and burdens) and their meaning structure, using an empirical phenomenological approach, with consideration of health behaviour change theories, we identified three second order constructs: 'disruption of habitual equilibrium', 'motivational mix of reassurance and risk', and 'curation of self efficacy'. Prior knowledge of the trial participants helped in the appreciation of their provinces of meaning.

### Burdens

#### Individual physical

The average age of the EDIPS trial participants at baseline was 57 years and most in this study sample were older (mean age 64). Many participants had pre-existing physical conditions, such as arthritis and hypertension and all were overweight or obese (BMI >= 25 kgm^-2^) at the start of the trial. The occurrence of setbacks, such as deteriorating physical condition or injury, was mentioned by many participants. Where people were struggling to maintain changes in difficult situations, setbacks were particularly unwelcome. The added burden could have led to any progress made being abandoned. Some participants had had medication for co-morbidities which caused problems,

*So that (medication) means I put weight on. Everything I eat goes into my weight*. (6: male, age 74, control)

#### Individual psychological

This was a major theme and the successful participants still had problems. Too much or the wrong type of food was a temptation, exercise seemed like a lot of effort and a previous bad experience or lack of ability was a deterrent.

*I went to the gym once and kinda thought it was terrible. I didn't want to go back *(8: male, age 72, intervention).

In some cases they became resigned to deteriorating health. If they compared themselves with someone who seemed to have had no regard for their own health, but nevertheless managed to stay slim and healthy, it seemed unfair that they were burdened with problems. Sometimes they were disheartened and struggled to maintain the effort.

It still doesn't make any difference, I am still not right. There seems to be

*no justice in it*. (3: female, age 66, control)

Some referred to media information, which might be an advertisement and thus considered untrustworthy, or to previous problems and confusion with mixed messages.

*There is always something coming up that you always thought was good for you and now they tell you that it isn't *(13: female, age 68, intervention).

#### Social

Many of the participants referred to the demands of their work and social situations. A number had caring responsibilities. The nature of caring responsibilities means that the demands vary and where the condition of the people being cared for is deteriorating the demands are likely to increase. One participant described how her husband's progressive dementia meant that, whereas at the start of the EDIPS she could go out running, later on she was restricted by having to stay in to look after him.

That was obviously the beginnings where he was reluctant to co-operate and things like that. But he was still concerned about me going out. (for a run) But I could go and I wasn't worried about leaving him at that point. He could still get out and about and use public transport so I could think about me that year, which was just as well. But it gradually sort of slid.... In the earlier day's stages of the programme where it wasn't quite so essential that I was there with (husband), I used to fit in swimming and other things instead of the Rosemary Connelly stuff. I could do something out of the house, a bit more active. Well, Tuesdays and Fridays when (Husband) is in his day centre, that's the days I go out and I either walk or I jog. (10: female, age 60, intervention)

Another participant described the care of her elderly mother.

*She has got me worn out at the minute I feel really tired and it is getting harder and harder to look after her. She is getting hard of hearing and I am having to repeat everything and repeat everything and she forgets (15: female, age 56, intervention)*.

Some people mentioned work issues and explained how work stress made change difficult whilst others referred to social relationships that hindered their ability to maintain lifestyle changes. There were examples where the person in charge of the family food was not the person directly receiving the intervention advice and some traditional views over portion sizes for men and an association of baking and comfort provision.

*Again my wife fails me there. She thinks that I should have more than her. She puts a small portion on her plate and a big one on mine. (7: male, age 67, intervention*)

There was some criticism of unhelpful or insensitive advice given by GPs.

*No disrespect to him like (GP), but all he said was, em, well just watch your diet and that was all I got *(3: female, age 63, control).

*All he can say to me at the moment is, "Come on lose the weight, you are going to end up like your mother". He annoyed us*. (15: female, age 56, intervention)

Looking back, many people were clear that they could not have undertaken major changes when their circumstances were different. Most of the people interviewed were retired and had time to devote to improving their lifestyle, but they were doubtful about the possibility of achieving behaviour change alongside work.

*Your health sort of gets pushed further away as your days get filled up with work as it were *(10: female, age 60, intervention).

*I think in life you can cope with so many things and then it just gets too much for you. You need to deal with some things before you can move on and deal with others*. (11: female, age 47, control)

Social occasions, such as Christmas festivities or parties and the associated food sometimes presented challenges.

*It was just nice to do a Sunday lunch cause the family was all there so it was nice to do that*. (11: female, age 47, control)

#### Environment

Some participants expressed concern about facilities. One person had difficulty walking and swimming was her best option, but she had transport problems getting to the pool. Another was concerned about the possible closure of a swimming pool.

*I love swimming. Since I was 14 I have had a problem with my right leg and it is the one thing that I can do well. I can't walk far and I can't run but I can swim*.

I am a bit worried in that Sunderland is talking about a new swimming pool and I am frightened, well concerned, that they will shut this one once the new one is built and it will probably be more like a leisure pool and you can't really swim in them. You can't get the speeds up you know. We usually find that it is best to go about three pm and then the lunch time rush is over and it is before the children come in from school. Sometimes when we go, especially when it is bad weather, there is just us two in the pool. In a way it is lovely but in another it is another nail in the coffin for closing it. I can't see two pools being run within a mile of each other. I can't see it happening. (9 female, age 57, control)

Another participant could have been discouraged from walking by a bad experience in her locality.

*It just made us more wary of where I was walking. Instead of going under the underpass, it's not very big but it's an underpass and since that happening [mugged] I have walked the main road way *(2: female, age 63, intervention).

Cost was a consideration for many. Even though the most common exercise of choice was walking there were still cost considerations.

*I get a pension off it......not very much mind – can't buy a new pair of shoes with it, that's for sure *(3: female, age 66, control)

The weather and seasons were regularly mentioned. Walking is often the cheapest and best exercise option, but poor weather can be a deterrent, especially for older people.

### Props

As expected with this group, who were selected for their ability to maintain behaviour change, this category was extensive. Stimulus control and substitution, (e.g. not buying tempting foods) reinforcement management (rewards) and helping relationships were emerging themes that were regularly mentioned and anticipated from the literature. Other categories also emerged from the data analysis.

#### Individual physical

Biological feedback, feeling better or feeling fitter after accomplishing changes, supported change maintenance.

*I just always feel good when I come out of the gym (15: female, age 56, intervention)*.

Being able to get the right professional help and in the example below, timely medication to help cope with a setback was important, especially where people had made the effort to change and this was being undermined by a temporary problem.

*The medicine was as a result of talking to your physiotherapist and she said that I should ask for an anti-inflammatory drug, which I did *(7: male, age 67, intervention).

#### Individual psychological

In general the tone of the interviews was positive. Problems were often cited to explain how they were able to overcome difficulties and develop strategies. For example where food was a temptation they might choose not to buy it.

Self efficacy was a theme which was expected from the literature and some participants expressed this clearly and directly.

*I have a lot of will power. So I knew I could do it when I started*. (2: female, age 63, intervention)

Previous success, such as with smoking cessation contributed to self efficacy and some people had already started to make diet changes previous to joining the EDIPS trial. For some talking to and encouraging themselves was important.

Well I have this little thing in me, I used to be fatter and now I'm thinner 'cause I have cut down my dinner, by a third. I would say now that it's a child's portion. (2: female, age 63, intervention)

Some had routines, such as an afternoon walk which they felt good about completing or rules for themselves about portion size which they were pleased to achieve. Some were clearly orientated to goals and others expressed a more general desire to stay on track or avoid ill health. Many mentioned the time it took, usually about two years, to absorb the changes and routines into their lifestyles.

Enjoyment of a new lifestyle, feeling pleased and rewarding themselves for maintaining a beneficial change was a strong theme. Many mentioned the satisfaction of achieving a goal, *['satisfied since you have done it'] *and the way it made them feel good to have accomplished a task, *['good to be alive']*. They took pride in staying on track. It was clear that in many cases the changes had become a way of life and part of their routine which they would miss if discontinued.

*I look forward to them [walks] now. It gives you time to reflect on different things as you walk... ...Yes, I would miss it and I think I would be restless to get out again (8: male, age 72, intervention)*.

The ways in which people monitored themselves, and gave themselves permission to deviate from the norm for special occasions or made allowances for changing burdensome circumstances were recurrent themes within the data. Most people set themselves rules or routines with allowances. This emphasised the ongoing nature of change maintenance and the quite complex systems people used to maintain an equilibrium or balance between best practice and realistically achievable changes.

*I think that target has pushed me or pulled me, or kept me going... ...where I might have slipped and thought I wont do this exercise today (10: female, age 60, intervention)*.

Many mentioned the impact of the original diagnosis of IGT as a motivating factor. Even after some years, the shock of finding out was still with them and they were usually able to recall a cautionary tale of a friend or relative, who had diabetes.

*He [GP] suddenly, out of the blue came out and said, "Well you know Mrs X you are glucose intolerant". Well I didn't know and nobody had told me till then, so I was taken quite by surprise you know. Once he told me about that I walked home in a daze, I was so amazed (3: female, age 66, control)*.

#### Social

Many referred to social relationships which helped in making and maintaining change Sometimes the new regime would be keenly adopted.

*So she [wife] takes an interest in what I have been told and whatever. It's good for us both, 'cause everything I do or am told to do she does *(8: male, age 72, intervention).

There was appreciation of the individual professional support and the way in which this had helped them to develop self regulation strategies. Intervention group participants appreciated the motivational interviewing process.

*I always feel guilty. Always, I think that she (dietician) will tell me off but she never does. She will just say "come on J what went wrong?" She will say "what went wrong?" She doesn't tell me off, but I always think I will get told off today for putting weight on. It is something in my head, but no she is lovely. We usually sit and reason it out and think well I did this or I had that. I went a bit overboard on something else you know and she would say, "What have you learnt from that?" and I will say well I shouldn't have done this and I shouldn't have done that. She gets you thinking about your food, thinking about what you eat and it's ...you know. I mean if you are going to come in thinking you are going to get wrong all the time, I mean you aren't going to come are you? We just analyse it out. It just gets you thinking about what you have done*. (15: female, age 56, intervention)

Some control group participants had been helped by their GP, referred to a dietician, or independently joined a weight loss group.

The timescale for professional support reflected the time needed to absorb lifestyle change and the timeliness of the intervention in relation to life course was a strong theme.

*I need somebody to keep me going, to keep me on track and make sure that I am not drifting off. I think that was the first year, but it probably rolled on into the second year*. (10: female, age 60, intervention)

The trial monitoring was sometimes expressed explicitly as a motivating factor that contributed to maintaining lifestyle change.

...*but, because I know that I have to come back in I have to stick to the rules so that you can check up to see if I am doing what I was told to do *(7: male, age 67, intervention).

The control group only attend once a year for their review, but monitoring may contribute to control group success. This was an important finding in this study.

*'Cause I get a good MOT every time I come here *(6: male, age 74, control).

Quite often participants would refer with pleasure to ways in which their lifestyle changes influenced people round them. For example how visiting grandchildren would eat fruit if that is what was available and would stop looking for biscuits, or a friend or partner might be persuaded to join the gym or go walking.

#### Environment

A stimulating environment, *['walks along the coast'] *and good facilities, [*'It is a heated pool and that would be better'] *encouraged exercise. Cost issues were mentioned. For example the newly introduced free bus passes helped in accessing facilities and made it easier for people to find nice places to walk.

## Discussion

### Main findings

In this qualitative study, nested within the EDIPS RCT, we aimed to develop an understanding of how people are able to maintain behaviour change. Maintenance themes, identified in organisational levels, (individual, social and environmental) were divided into those that help and those that hinder behaviour change maintenance, ('props' and 'burdens'). The ongoing nature of props and burdens, the way they alter over time and the effect this has on people's ability to maintain lifestyle improvements were important findings. There were positive changes, such as improved social circumstances, feeling better, or becoming fitter, and setbacks, such as injury, or the closure of an exercise facility. These changes impacted on people's ability to achieve their behavioural aims.

Organisational levels in health promotion theory were described by Kelly et al[26], and a similar structure was used by Dahlgren et al, to acknowledge wider determinants of health and socioeconomic influences [27]. Social cognitive theory acknowledges the importance of the environment and the social circumstances of an individual, and the effects these have on individual values in a triadic reciprocality described by Bandura as reciprocal determinism [28].

Many successful participants had developed sophisticated systems of rewards and self-monitoring, permissions and allowances, stimulus control and substitution, and the ability to talk to and encourage themselves, persuading, warning and congratulating themselves. These behavioural strategies were expected from the literature [18,29].

The three novel second order constructs we identified have a particular relevance to diet and exercise behaviour change. These second order constructs, which could have a practical application in health behaviour intervention planning, are: disruption of habitual equilibrium (with the continuous and variable nature of props and burdens), motivational mix of reassurance and risk (with the diagnosis of IGT as a less emotive condition), and curation of self efficacy (with timeliness and individualised success criteria).

In our analysis the word 'burden' was used specifically (as opposed to barrier which is usual in the literature) to convey the ongoing nature of the problems encountered by participants. Physical ill health (such as arthritis and hypertension), and social responsibilities (such as caring for an elderly relative) were both continual and variable. In this respect, diet and exercise behaviour change was identified as different from some other behaviour change paradigms (for example smoking cessation). Healthy eating and increasing activity require continual proactive effort. Even when the beneficial change had become habitual, setbacks (such as physical injury or greater care demands) could disrupt this equilibrium. To conceptualise this we identified a second order construct: 'disruption of habitual equilibrium'.

Evidence statements in the recently proposed NICE guidance on behavioural change interventions accept the variety of components in psychological models of health behaviours and identify some components as specific to one theory, and others as shared across different theories [17]. In this qualitative work we have identified key principles of behaviour change that are described in the health behaviour models we reviewed, either specific to one model or shared across models [30].

The impact of IGT diagnosis was often mentioned, even though this had occurred three to five years previously. The diagnosis knowledge would be described according to the Health Belief Model (HBM) as a threat or cue to action. However the diagnosis of IGT was viewed by participants as both a risk and a reassurance (i.e. not diabetes yet), and therefore this diagnosis may contribute to different aspects of perceived controllability as described in Leventhal's self-regulation theory (sometimes called the Common Sense Model) [31]. Participants recognised IGT as a condition with an 'identity, cause and consequence', as described by Leventhal, but the 'emotional response and fear' was more specifically associated with diabetes. Self regulation in Leventhal's model is seen as a dynamic process where people monitor their coping strategies, and persist with those that are successful. Avoiding diabetes was a strong motivator. The effect of the diagnosis and monitoring are particularly important in considering control group success. External reinforcement through the clinical monitoring may support self regulation so that the rewarded changes become habitual. The intervention group had the additional support of the motivational interviewing experience. To conceptualise the effect of diagnosis and monitoring we identified another second order construct: 'motivational mix of reassurance and risk'

The intervention in the EDIPS trial was based on the trans-theoretical model (TTM) which has a temporal dimension, the stages of change construct. This qualitative study referred specifically to participants in the maintenance stage, so consideration of stage progression was not relevant. However time issues were a recurrent theme, both the timeliness of the intervention opportunity coincident with the readiness of the participant, and the time it took for lifestyle changes to be absorbed and to become habitual. In the TTM maintenance stage the behavioural processes are identified as counter-conditioning, forming helping relationships, reinforcement management and stimulus control, with components of decisional balance, self-efficacy, and temptation.

To determine 'success' in this study we norm referenced individual beneficial change relative to baseline maintained for more than one year, and selected the most successful in the three categories, (activity increase, calorie and fat consumption reduction). In general the participants perceived themselves as successful (although they were not told they had been selected as such). Such perceptions of success may be motivational. Perception of success as a motivator links with the concept of self efficacy in Social Cognitive Theory (SCT) [17] and in the TTM. Maintaining diet and exercise change required continual pro-active effort; therefore the tenet of SCT that self belief can inform subsequent performance was particularly relevant. The Theory of Planned Behaviour (TPB) has a similar dimension of perceived behavioural control [17]. There were plenty of instances where participants expressed enjoyment from the new regime and feeling pleased with themselves for having made changes. Even the tone of the interviews was very positive. To conceptualise the effect of this perception of success, we identified another second order construct: 'curation of self efficacy.' Issues relating to the timeliness of intervention, the time it takes for changes to be established as routine, and the methodology of a motivational interviewing approach all contributed to this construct.

Imitation and modelling, the idea that human behaviour can be learnt from observing others, are features of SCT. The intervention did include cook and eat sessions and a newsletter which participants contributed to, although most of the intervention was delivered on an individual basis. Some of our successful participants described how they were keen to influence friends and relatives with their new lifestyle regimes.

### Strengths and limitations

This is one of the first qualitative studies undertaken alongside an RCT of diabetes prevention and highlights the utility of data available from trial participants. The long timescale of the EDIPS, with regular collection of individual behavioural data, provided a valuable opportunity as the participants had quantifiable experience of maintaining behaviour change over at least two years. As the findings are based on a relatively small sample, caution must be exercised in generalising the findings beyond the study group.

### Implications

Our selection criteria for success were based on individual percentage beneficial change compared with baseline. For example if someone had reduced their fat intake from 40% to 35% (calorie equivalent) for two years they would be identified as successful, even though they had not achieved the recommended 30% level specified in the EDIPS trial protocol. From a behavioural perspective, in promoting self efficacy and perceived control, there were positive implications resulting from this definition of success. In terms of translating behavioural process measures to health outcomes there is the opportunity to analyse the EDIPS trial data in further research investigating the utility of individual percentage beneficial change as an intervention target. For intervention development it may be better to start with small changes and factor in success using individually referenced criteria to promote self efficacy for further change.

The impact of the diagnosis of IGT, with the motivational mix of reassurance and risk, and the regular clinical monitoring may contribute to implementation and maintenance of beneficial health behaviour strategies. In line with Leventhal's model the 'label' IGT could contribute to the development of self regulation. Monitoring was particularly relevant in considering control group success, whereas in the intervention group there was the additional positive feedback from the intervention team.

The data suggest that it takes time for behaviour change to be learnt and absorbed into routine. This is in line with social cognitive theory where 'the practice of behaviour change leads to skill development'. Interventions should provide sufficient time for participants to learn and practice change maintenance and monitoring skills. It is worth considering longer term but less intensive behaviour change interventions for wider implementation, as these may be more effective and cost-effective in the long run. This requires comparative evaluation and may be amenable to meta-analysis of published trial data.

In this study we identified the ongoing and variable nature of burdens that hinder change maintenance, the importance of the participants' physical condition, and the value of timely assistance to ameliorate the impact of change in physical health. Pain or discomfort is a deterrent and the memory of this can last. Access to various exercise options helps to pre-empt problems, for example swimming if walking is problematic. There are resource implications to providing advice on and access to a range of support and facilities. Positive physical feedback (e.g. being fitter or healthier) is an encouragement, but it takes time for this to be appreciated. Participants need to be prepared for both positive and negative physical feedback.

The influence of social relationships is important and consideration should be given to involving significant others. For example, if a participant's meals are prepared by someone else, then it makes sense to include that person in change strategies. People who have developed successful strategies may be able to contribute to peer group success as role models for imitation and modelling.

The EDIPS trial intervention was based on individual motivational interviewing. This intervention approach has proved both effective and efficient in diabetes prevention studies. [12] However, an effective public health diabetes prevention strategy will include long term population-based interventions as well individually targeted interventions. As we had the opportunity to consider qualitative findings in relation to trial data we could elucidate ways in which the trial intervention might be adapted and improved, whilst retaining the basic intervention method for which there is the strong evidence base.

## Conclusion

Our constructs of props and burdens and the concept of 'disruption of habitual equilibrium' facilitate an holistic view of the person within their context and provide a tool that could be tested for intervention planning and review.

This qualitative study nested within a diabetes prevention trial has allowed consideration of lifestyle change from the perspective of participants who have successfully maintained behaviour change. Their experiences are useful in highlighting the significant challenges in planning the wider implementation of diabetes prevention strategies for health related behaviour change.

## Competing interests

The authors declare that they have no competing interests.

## Authors' contributions

The research reported here was undertaken by LP and supervised by MW and SMM in partial fulfilment of the degree of MSc in Health Sciences at Newcastle University. All authors met regularly to discuss the transcript data, topic guide development, coding concepts and development of the themes. MW and SMM reviewed, contributed to and revised the draft manuscript prepared by LP. All authors read, and agreed the final version.

## Pre-publication history

The pre-publication history for this paper can be accessed here:



## References

[B1] Campbell M, Fitzpatrick R, Haines A, Kinmonth AL, Sandercock P, Spiegelhalter D, Tyrer P (2000). Framework for design and evaluation of complex interventions to improve health. BMJ.

[B2] Nutbeam D (1998). Evaluating health promotion - progress problems  and solutions. Health Promotion International.

[B3] Nutbeam D (1999). The challenge to provide 'evidence' in health promotion. Health Promotion International.

[B4] Moffatt S, White M, Mackintosh J, Howel D (2006). Using quantitative and qualitative data in health services research - What happens when mixed method findings conflict? [ISRCTN61522618]. BMC Health Services Research.

[B5] Astrup A (2004). The effect of exercise and diet on glucose intolerance and substrate utilization?. Nestle Nutr Workshop Ser Clin Perform Programme.

[B6] Costacou T, Mayer-Davis E.J (2003). Nutrition and prevention of type 2 diabetes. Annual Review of Nutrition.

[B7] Pedersen BK, Saltin B (2006). Evidence for prescribing exercise as therapy in chronic disease. Scandinavian Journal of Medicine & Science in Sports.

[B8] Health D, Department of Health (2001). National Service Framework for Diabetes.

[B9] Knowler WC, Barrett-Connor E, Fowler SE, Hamman RF, Lachin JM, Walker EA, Nathan DM, Diabetes Prevention Program Research G (2002). Reduction in the incidence of type 2 diabetes with lifestyle intervention or metformin.. N Engl J Med.

[B10] Pan XR, Li GW, Hu YH, Wang JX, Yang WY, An ZX, Hu ZX, Lin J, Xiao JZ, Cao HB, Liu PA, Jiang XG, Jiang YY, Wang JP, Zheng H, Zhang H, Bennett PH, Howard BV (1997). Effects of diet and exercise in preventing NIDDM in people with impaired glucose tolerance. The Da Qing IGT and Diabetes Study.. Diabetes Care.

[B11] Tuomilehto J, Lindstrom J, Eriksson JG, Valle TT, Hamalainen H, Ilanne-Parikka P, Keinanen-Kiukaanniemi S, Laakso M, Louheranta A, Rastas M, Salminen V, Uusitupa M, Finnish Diabetes Prevention Study G (2001). Prevention of type 2 diabetes mellitus by changes in lifestyle among subjects with impaired glucose tolerance.. N Engl J Med.

[B12] Penn Linda, White Martin (2008). Interventions to prevent or delay the onset of type 2 diabetes in people with impaired glucose tolerance. BMJ Health Intelligence, Public Health.

[B13] Eriksson J, Lindstrom J, Valle T, Aunola S, Hamalainen H, Ilanne-Parikka P, Keinanen-Kiukaanniemi S, Laakso M, Lauhkonen M, Lehto P, Lehtonen A, Louheranta A, Mannelin M, Martikkala V, Rastas M, Sundvall J, Turpeinen A, Viljanen T, Uusitupa M, Tuomilehto J (1999). Prevention of Type II diabetes in subjects with impaired glucose tolerance: the Diabetes Prevention Study (DPS) in Finland. Study design and 1-year interim report on the feasibility of the lifestyle intervention programme. Diabetologia.

[B14] Rollnick S, Mason P, Butler C (1999). Health Behaviour Change.

[B15] Adolfson (2002). Treating obesity: a qualitative evaluation of a lifestyle intervention for weight reduction. Health Education Journal.

[B16] Frandsen KB, Kristensen JS (2002). Diet and lifestyle in type 2 diabetes: The patient's perspective. Practical Diabetes International.

[B17] Rennie T, Health D, David Taylor, Michael Bury, Natasha Campling, Sarah Carter, Sara Garfied, Jenny Newbould (2006). A Review of the use of the Health Belief Model (HBM), the Theory of Reasoned Action (TRA), the Theory of Planned Behaviour (TPB) and the Trans-Theoretical Model (TTM) to study and predict health related behaviour change.. DOH (UK) NICE guidance.

[B18] DOH , Jepson R, Harris F, MacGillivray S, Kearney N, Rowa-Dewar N (2006). A review of the effectiveness of interventions, approaches and models at individual, community and population level that are aimed at changing health outcomes through changing knowledge attitude and behaviour. DOH (UK) NICE guidance.

[B19] Wing RR, Hill JO (2001). Successful weight loss maintenance. Annual Review of Nutrition.

[B20] Mclean H (1991). Patterns of diet related self-care in diabetes. Social Science and Medicine.

[B21] Peel E, Parry O, Douglas M, Lawton J (2005). Taking the biscuit? A discursive approach to managing diet in type 2 diabetes. Journal of Health Psychology.

[B22] Ritchie J, Lewis J (2003). Qualitative research practice: a guide for social science students and researchers.

[B23] QSR International Pty Ltd (2006). NVivo qualitative data analysis software; Version 7.

[B24] Silverman D (2000). Doing Qualitative Research : a practical handbook.

[B25] Patrick A (2004). Empirical Phenomenology. An Approach for Qualitative Research. Papers in Social Research Methods LSE Methodology Institute.

[B26] Kelly MP, Charlton BG, Hanlon P (1993). The four levels of health promotion: An integrated approach. Public Health.

[B27] Dalgren G, Whitehead M (1991 ). Policies and strategies to promote social equity in health.

[B28] Bandura A (2004). Health promotion by social cognitive means. Health Educ Behav.

[B29] Klem ML, Wing RR, Lang W, McGuire MT, Hill JO (2000). Does weight loss maintenance become easier over time?. Obesity Research.

[B30] Noar SM, Zimmerman RS (2005). Health Behavior Theory and cumulative knowledge regarding health behaviors: are we moving in the right direction?. Health Education Research.

[B31] Marteau TM, Weinman J (2006). Self-regulation and the behavioural response to DNA risk information: A theoretical analysis and framework for future research. Soc Sci Med.

